# Reflecting on the relationship between residential schools and TB in Canada

**DOI:** 10.5588/ijtld.22.0371

**Published:** 2022-09-01

**Authors:** C. Heffernan, G. Ferrara, R. Long

**Affiliations:** 1 Tuberculosis Program Evaluation and Research Unit, Department of Medicine, University of Alberta, Edmonton AB, Canada; 2 Division of Pulmonary Medicine, Department of Medicine, University of Alberta, Edmonton, AB, Canada; 3 School of Public Health, University of Alberta, Edmonton, AB, Canada

Canada’s residential schools were boarding institutions for Indigenous children that operated between 1831 and 1996, first as missionary schools run by churches supported by federal aid, then, post-confederation, as policy and means of providing an “education” (see Figure). In practice, though, they served as a (forceful) mechanism to assimilate Indigenous children into the newly dominant society of Europeans.^1^,^2^ The residential school system has had negative enduring effects on individuals, communities and Canadian society overall. As a result of these harms, the largest class action settlement in Canada’s history was achieved in 2007: the Indian Residential Schools Settlement Agreement.^3^ As an element of this settlement, a Truth and Reconciliation Commission (TRC) was convened to provide a historical account of experiences (i.e., documentation of the truth) of children in the residential school system. This was done by survivors, who bravely recounted being separated from family, being dispossessed from land, culture and language, and experiencing other psychological, physical and sexual forms of abuse, including withholding food as punishment and experiments in malnourishment.^4^–^8^ Of note, 3,200 children were confirmed to have died as wards of these schools – at a rate far higher than school-aged children in the general population.^4^,^8^ Significant gaps in data, however, mean many remain unidentified, that deaths are probably more numerous than reported, and that undiscovered burial sites likely exist. Consequently, one full report from the TRC is titled, “*Missing Children and Unmarked Burials*”.

**Figure:**
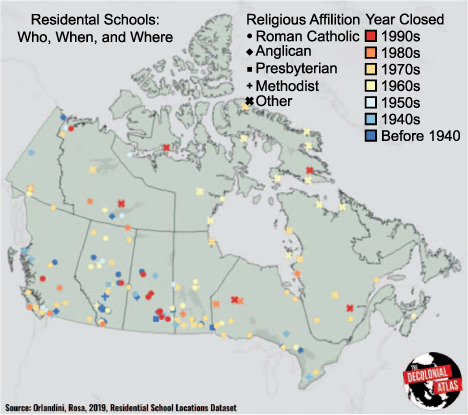
Map of residential schools by affiliation and years of operation. *Prairies are Alberta (bottom row = second from the Westernmost Province), Saskatchewan (bottom row = third from the Westernmost Province) and Manitoba (bottom row = fourth from the Westernmost Province). Reproduced from the Decolonial Atlas; free Decolonial Media Use License 0.1.^23^

Indeed, at the time of writing this article (May 2022), Canada is confronting the one year anniversary of the recovery of remains in an unmarked grave outside the site of a former residential school in Kamloops, British Columbia.^9^ This recovery was the first of many across the nation. Although age and cause of death among those buried have yet to be established, contemporaneously gathered evidence implicates a role for TB.^10^ Theprovenanceof these graves is not fully understood, but their existence has reopened wounds relating to the “missing children” of the residential school system as well as to the persistent inequitable distribution of TB disease in Canada today. In addition to the aforementioned abuses, conditions in the schools were such that disease and death among the children was unmanageable and included the spread of smallpox, measles, influenza and TB.^10^,^11^ The latter – epidemic TB – is especially troubling because, compared to other acute viral outbreaks, it unfolds more slowly. The historical records support many missed opportunities to intervene, and a general apathy to the wellness of these children. In fact, the dire experience of TB disease within residential schools in the Prairie Provinces of Canada was documented by Dr Peter Henderson Bryce, the Chief Medical Officer of health for the Department of Indian Affairs at the time.^10^ Bryce’s health surveys in the early 1900s revealed horrific rates of TB deaths in residential schools. He identified a single school in southern Saskatchewan where 69% of students had perished either while attending or shortly thereafter, the majority of whom succumbed to TB.^10^ Bryce clearly described that the intrinsic risk of TB within the schools arose from a confluence of factors, including the fact that 1) many facilities were re-purposed buildings featuring poor ventilation; 2) they were overcrowded; 3) minimal efforts were undertaken to isolate sick children and staff; 4) children, already at increased risk for progression to disease, were rendered yet more vulnerable by stress and malnourishment; and 5) there was limited access to medical care for TB screening or management of other viral illnesses (see summary in the Table). These findings were paired with requests to finance improvements to facilities.^12^ Corrective measures were, however, considered too costly, and outside the mandate for the provision of “education”, and his pleas were ultimately unheeded. Shortly thereafter, funding to sustain health surveys at residential schools was cut and Bryce’s post ended in 1921. In 1922, he published a scathing critique of federal inaction titled, *“A National Crime: an Appeal for Justice to the Indians of Canada”*.^12^ As predicted by Bryce, without interventions to improve conditions, an overall TB mortality rate of 8,000/100,000 population in the residential school system was seen in the 1930s compared to rates of 51–79/100,000 population in the country overall for the same decade.^13^,^14^

**Table:** Summary of risks for infection by Mycobacterium tuberculosis, and disease if infected

Risk of infection	Risk of disease after infection
Entering school with infection/disease Children who were already ill were not to be admitted but may have been for purposes of reaching enrolment quotas (funding to the schools was on a per capita basis) Lack of access to medical care Re-purposed buildings with characteristics related to: Overcrowding Poor ventilation; poor ultraviolet light exposure	Repeated infections/infecting dose Malnutrition/undernourishment diet type; withholding of food as punishment Stress-induced dislocation from family and home; forbidden use of language, cultural practice, and religious practices; abusive punishments and practices Child labour Communication barriers Concurrent viral illness Lack of access to medical care

It is now the centenary of the document, *A National Crime*, and a demonstrable unfairness in the distribution of TB persists. To this day, Indigenous peoples who comprise less than 5% of the total Canadian population experience the highest overall rate of disease.^15^ Moreover, the Prairie Provinces of Canada remain a regional hotbed with ~50% of the total annual episodes of TB disease being diagnosed in this population.^16^ At the same time, the overall rate of disease among non-Indigenous Canadians is approaching pre-elimination at 0.4/100,000 population.^15^ Taken together, these facts suggest that Canada – a nation with the resources to achieve TB elimination – suffers from a lack of political will at every level of the government to prevent occurrence of the disease among all citizens equally, and therein lies the rub. Although residential schools are no longer operating as physical amplifiers of disease, prevailing colonialist attitudes uphold systemic exclusion of Indigenous peoples from full societal participation.^2^,^17^ Measures to close political and economic welfare gaps are consistently viewed as out of scope, are slow to start and tend to be underfunded. Great needs, as they relate to TB, include the recognition of the intergenerational traumatic effects of residential schools leading to justice involvement and over-incarceration of Indigenous peoples, poverty reduction, addressing housing issues (crowding, poor ventilation), lack of access to clean water and food insecurity.^18^ More importantly, though, is that any government that promises to tackle Indigenous issues in Canada needs to specify the mechanisms for being held to account for their implementation, or failure, because mistrust due to a chronic lack of action is a barrier to success.

Independent of systems-level change and governmental action, TB programmes in Canada ought to acknowledge and confront the legacy of residential schools as relevant to the experience of disease for Indigenous peoples and communities. Promising steps are being taken to centre Indigenous peoples’ experience and knowledge in the provision of such services. In March 2022, the 8^th^ Edition of the Canadian Tuberculosis Standards was published and it newly featured a chapter authored by representatives from each of the three culturally and ethnically distinct groups of Indigenous peoples in Canada: First Nations, Métis and Inuit.^19^ Another new addition to these Standards promotes monitoring and evaluation of the integration of best practices into programmes and for associated reports to be made public as a means of building bridges of trust to underserved, equity-seeking, groups.^20^ Finally, a multi-year project, funded by the Canadian Institutes of Health Research signature initiative, Pathways to Health Equity for Indigenous Peoples, provides Indigenous communities on the Prairies with surveillance data, and therefore their own epidemiological experience of TB disease that, in turn, generates community-identified and led solutions.^21^,^22^ A sustained biosocial, all-stakeholder and community-centred response is needed to reverse disease disparity and diminish the impact of the failures of residential schools to Indigenous peoples of Canada.
